# *Salix purpurea* Stimulates the Expression of Specific Bacterial Xenobiotic Degradation Genes in a Soil Contaminated with Hydrocarbons

**DOI:** 10.1371/journal.pone.0132062

**Published:** 2015-07-10

**Authors:** Antoine P. Pagé, Étienne Yergeau, Charles W. Greer

**Affiliations:** 1 Department of Natural Resource Sciences, McGill University, Montréal, Québec, Canada; 2 Energy, Mining and Environment, National Research Council Canada, Montréal, Québec, Canada; NERC Centre for Ecology & Hydrology, UNITED KINGDOM

## Abstract

The objectives of this study were to uncover *Salix purpurea*-microbe xenobiotic degradation systems that could be harnessed in rhizoremediation, and to identify microorganisms that are likely involved in these partnerships. To do so, we tested *S*. *purpurea*‘s ability to stimulate the expression of 10 marker microbial oxygenase genes in a soil contaminated with hydrocarbons. In what appeared to be a detoxification rhizosphere effect, transcripts encoding for alkane 1-monooxygenases, cytochrome P450 monooxygenases, laccase/polyphenol oxidases, and biphenyl 2,3-dioxygenase small subunits were significantly more abundant in the vicinity of the plant's roots than in bulk soil. This gene expression induction is consistent with willows' known rhizoremediation capabilities, and suggests the existence of *S*. *purpurea*-microbe systems that target many organic contaminants of interest (i.e. C4-C16 alkanes, fluoranthene, anthracene, benzo(a)pyrene, biphenyl, polychlorinated biphenyls). An enhanced expression of the 4 genes was also observed within the bacterial orders *Actinomycetales*, *Rhodospirillales*, *Burkholderiales*, *Alteromonadales*, *Solirubrobacterales*, *Caulobacterales*, and *Rhizobiales*, which suggest that members of these taxa are active participants in the exposed partnerships. Although the expression of the other 6 marker genes did not appear to be stimulated by the plant at the community level, signs of additional systems that rest on their expression by members of the orders *Solirubrobacterales*, *Sphingomonadales*, *Actinomycetales*, and *Sphingobacteriales* were observed. Our study presents the first transcriptomics-based identification of microbes whose xenobiotic degradation activity in soil appears stimulated by a plant. It paints a portrait that contrasts with the current views on these consortia's composition, and opens the door for the development of laboratory test models geared towards the identification of root exudate characteristics that limit the efficiency of current willow-based rhizoremediation applications.

## Introduction

The contamination of soils with hazardous hydrocarbons (i.e. alkanes, aromatics, organochlorides) raises growing concerns for public health worldwide [1–3], which feeds a push for the development of better mitigation technologies. Among the emergent approaches that promise to produce such tools, phytoremediation distinguishes itself with a remarkably high benefit/cost ratio. Indeed, rhizosphere phytoremediation (rhizoremediation), can promote the degradation of many common hydrocarbon contaminants [4–6], including some that are notoriously recalcitrant (e.g. pyrene, phenanthrene, anthracene, pentachlorophenol, polychlorinated biphenyls) [7–9], and its usage only requires modest financial investments. Yet, the technology's efficiency remains too low to meet many modern soil cleanup needs (e.g. rapid spill mitigation and decommissioning of brownfields for repurposing projects). To increase its commercial competitiveness, it has to be faster.

The rhizoremediation of organic contaminants is mainly based on certain plants’ ability to increase microbial xenobiotic degradation rates [10–14]. Like most plant modifications of microbial functions in soils [15–17], these alterations are accomplished through root exudation [18–21]. Yet, proficient exudates appear sometimes ill formulated, and/or delivered in insufficient quantities, to achieve optimal microbial stimulations [19, 22–24]. As this likely limits the efficiency of many rhizoremediation applications, relieving these bottlenecks could globally improve the technology’s applicability. Selective breeding and/or direct genetic manipulations would probably enable the necessary physiological adjustments, as they have proven their worth in similar endeavors [25, 26]. However, the success of this bioengineering would obviously hinge on a precise understanding of the exudate characteristics that hinder current applications, which remain poorly constrained at this time. The development of laboratory models that reproduce solicited plant-microbe xenobiotic degradation systems would likely fill many knowledge gaps, but it requires a prior identification of the microorganisms that participate in these partnerships. Unfortunately, studies that have specifically singled out such consortia remain scarce [27–32].

Willows (*Salix* spp.) possess a particularly great potential for the development of commercially competitive hydrocarbon rhizoremediation applications. Not only do they naturally improve the degradation of many such contaminants in soil [8, 9, 33–38], but they also tolerate high concentrations of phytotoxic compounds [36, 37], develop extensive root systems that have wide radiuses of influence [38], and have habitat ranges that cover many climatic zones [39]. As the aforementioned capabilities clearly rest on exudate-stimulated microbial xenobiotic degradation [12, 14, 28, 31], they are prime candidates for a laboratory-based enhancement. However, work conducted towards the identification of contributing microorganisms remains limited to the isolation of PCB (polychlorinated biphenyl)-degrading *Rhodococcus*, *Pseudomonas*, *Sphingobacterium*, and *Burkholderia* species [12, 28, 31]. Here, we carried out metatranscriptomic surveys to explore *S*. *purpurea*'s microbially assisted rhizoremediation capabilities, and to specifically pinpoint microorganisms whose activity is at the heart of the associated xenobiotic degradation systems.

## Materials and Methods

### Summary of previously conducted greenhouse rhizoremediation experiment

Approximately 240 L of contaminated soil was collected from the site of a dismantled petrochemical factory in Varennes (QC, Canada), a suburb of Montréal, and homogenized [14]. A permission to access the site and conduct the study was granted by Pétromont Inc. Decades of industrial activity (1953–2008) resulted in the accumulation of petroleum hydrocarbons and PCBs in an area to the southwest of the former factory, and peak concentrations of 4200.0 mg/kg total petroleum hydrocarbons (TPHs), 91.1 mg/kg polyaromatic hydrocarbons (PAHs), and 1.1 mg/kg total PCBs can still be measured in the field. However, the collected soil contained lower concentrations of all three contaminant categories (1317 mg/kg TPHs, 75 mg/kg PAHs, and 0.3 mg/kg total PCBs) after homogenization. This soil was distributed into twelve 20 L pots, 6 of which received *S*. *purpurea* cultivar Fish Creek plants that had previously been grown from cuttings in sterile potting soil for 8 weeks. The 12 pots were placed in a greenhouse maintained at 18–20°C for 6 months, during which they were watered frequently to maintain soil moisture near field capacity. Five samples (approximately 2 g each) were then collected from each of the 6 control (bulk soil) and 6 planted (rhizosphere soil) pot, they were combined and homogenized thoroughly into one 10 g sample per pot, and total RNA was extracted from 2 g of each homogenized sample with MoBio's RNA PowerSoil total RNA isolation kit (MoBio, Carlsbad, CA, USA). DNA was removed from the extracts with Ambion's TURBO DNAse (Life Technologies, Burlington, ON, Canada), and the digestion was confirmed by PCR with universal 16S rRNA gene primers. Metatranscriptomic sequencing datasets were generated with each one (i.e. one metatranscriptome per pot), following rRNA subtractions, the addition of a control RNA molecule, and the production of cDNA sequencing libraries, using an Illumina HiSeq 2000 instrument. Raw sequence data was cleaned and partitioned into rRNA and mRNA datasets with MG-RAST [40]. The details of these procedures are described in Yergeau et al. [14].

### Identification of oxygenase transcripts in metatranscriptomic datasets

The quality-controlled messenger RNA data associated with the 12 metatranscriptomes was gathered from MG-RAST (https://metagenomics.anl.gov/ IDs 4512575-4512580 for bulk soil and 4512587-4512592 for rhizosphere soil) in order to assess the level of expression of microbial oxygenase genes that are markers of selected biodegradation processes (Table 1). These genes code for enzymes that almost all had known substrates among the contaminants detected in the soil used for the greenhouse experiment [14]. Yet, it also included genes encoding for oxygenases whose substrates were likely present, but not directly assessed (e.g. benzoate 1,2-dioxygenase). The transcripts' nucleotide sequences were first translated in all 6 reading frames using the EMBOSS’ transeq program (http://emboss.toulouse.inra.fr/cgi-bin/emboss/help/transeq). The standard code table was used in order to account for the potential contribution of prokaryotes and eukaryotes to the generated metatranscriptomes. Putative protein-coding portions of the resulting amino acid sequences (i.e. sections bracketed by start and/or stop codons) were then collected with a custom script, and searched for homology with sequences of selected protein families obtained from the Fungene 7.4 repository [41]. The latter included: methane monooxygenase component A alpha chain (*mmoX*), alkane 1-monooxygenase (*alkB*), cytochrome P450 monooxygenase (*p450*), benzoate 1,2-dioxygenase subunit alpha (*benA*), naphthalene dioxygenase (*npah*), aromatic ring hydroxylating dioxygenase (*ppah*), laccase/polyphenol oxidase (*ppo*), biphenyl 2,3-dioxygenase large subunit (*bphA1*), biphenyl 2,3-dioxygenase small subunit (*bphA2*), and dibenzofuran 4,4a-dioxygenase large subunit (*dbfA1*). To reduce computing time, homology searches included a preliminary screening for 5mers matching any phylotypes of the selected amino acid databases. This procedure was conducted with blat [42], using the following parameters (-tileSize = 5-minMatch = 1-minScore = 0). Retained sequences were then searched using the hmmsearch program of package HMMER 3.0 [43] (E value 1e-8), and profile Hidden Markov Models (pHMMs) also obtained from the Fungene repository. Gene-specific transcript abundances were then tallied for each metatranscriptome, and corrected to account for over evaluations cause by the presence of the same gene on paired sequences.

**Table 1:** Selected marker genes. Families of oxygenase-related proteins that were used in this study, and their substrates that are relevant to the characteristics of the collected contaminated soil.

Genes	Protein families	Substrates	References
Petroleum hydrocarbons			
*mmoX*	methane monooxygenase component A, alpha chain	C1-C10 alkanes*, phenanthrene, anthracene, fluorene	[44, 45]
*alkB*	alkane 1-monooxygenase	C5-C16 alkanes*	[44, 46, 47]
*p450*	cytochrome P450 monooxygenase	C4-C16 alkanes*, fluoranthene	[44, 46–48]
*benA*	benzoate 1,2-dioxygenase subunit alpha	benzoate*	[49]
*npah*	naphthalene dioxygenase	acenaphthylene, anthracene, benzo(a)pyrene, ethylbenzene*, fluorene, 1-methylnaphthalene, 2-methylnaphthalene, naphthalene	[48, 50, 51]
*ppah*	aromatic ring hydroxylating dioxygenase	BTEX*, acenaphthylene, anthracene, benzo(a)pyrene, phthalate, benzoate*,fluorene, 1-methylnaphthalene, 2-methylnaphthalene, naphthalene, pyrene, etc	[52]
*ppo*	laccase/polyphenol oxidase	anthracene, benzo(a)pyrene	[53]
*bphA1*	biphenyl 2,3-dioxygenase large subunit	biphenyl*	[54]
*bphA2*	biphenyl 2,3-dioxygenase small subunit	biphenyl*	[54]
Organochlorides			
*mmoX*	methane monooxygenase	PCB congeners	[45]
*ppah*	aromatic ring hydroxylating dioxygenase	PCB congeners, chlorobenzenes	[52]
*bphA1*	biphenyl 2,3-dioxygenase large subunit	PCB congeners	[55, 56]
*bphA2*	biphenyl 2,3-dioxygenase small subunit	PCB congeners	[55, 56]
*dbfA1*	dibenzofuran 4,4a-dioxygenase large subunit	chlorinated dibenzofuran*	[57]

*Benzoate, biphenyl, benzene-toluene-ethylbenzene-xylene (BTEX), and chlorinated dibenzofuran are commonly observed in contexts similar to that of this study, but analyses enabling their detection weren’t carried out. C10-C50 total petroleum hydrocarbons were detected but C1-C16 alkanes weren’t measured individually.

### Taxonomic assignment of identified oxygenase transcripts

Transcripts whose protein-coding portions showed sequence homology with the selected oxygenase protein families were further analyzed to reveal the taxonomic identity of their parent microorganisms. The nucleotide sequences were searched against nucleotide versions of the Fungene databases described above, with blastn (http://blast.st-va.ncbi.nlm.nih.gov/Blast.cgi) (E value 1e-8), and taxonomic qualifiers were assigned to each one using a custom script implementing a "lowest common ancestor" rule (i.e. closest shared taxonomic level). Parameters were as follows: minimum bitscore = 80, minimum match above minimum bitscore = 1, top 5% bitscores considered. Gene- and taxa-specific transcript abundances were tallied for each metatranscriptomes, and again corrected for over evaluations resulting from the presence of the same gene on paired sequences. When distinct taxonomic identities were assigned to paired sequences that accounted for the same gene, qualifiers were assigned using the lowest common ancestor rule described above.

### Statistical comparisons of oxygenase expression levels in bulk and rhizosphere soil

Community-wide and taxonomically resolved oxygenase transcript abundances were adjusted to those of the RNA control for each sample, resulting in normalized values (transcripts per g of soil). Taxonomic data was then summed at the level of orders to facilitate statistical comparisons. *S*. *purpurea*’s influence on the collective expression of the selected gene set was then verified, both at the community and order levels, through ordination analyses conducted using the non-metric multidimensional scaling (NMDS) method implemented by the metaMDS function of the R package VEGAN [58]. The plants’ influence on the expression of each gene and gene-taxon combination was then evaluated individually. Bulk and rhizosphere soil transcript abundances were tested for normality and homogeneity of variance using the shapiro.test and bartlett.test functions implemented by R version 3.0.2 (http://www.r-project.org). When parametric assumptions were met, datasets were compared with T-tests using R's t.test function. When they were not, the data was log transformed, retested for normality and homogeneity of variance, and either compared with T-tests or with Wilcoxon Signed-Rank tests conducted with R's wilcox.test function.

## Results

### Influence of *S*. *purpurea* on the microbial community-wide expression of oxygenase genes

**Fig 1:**
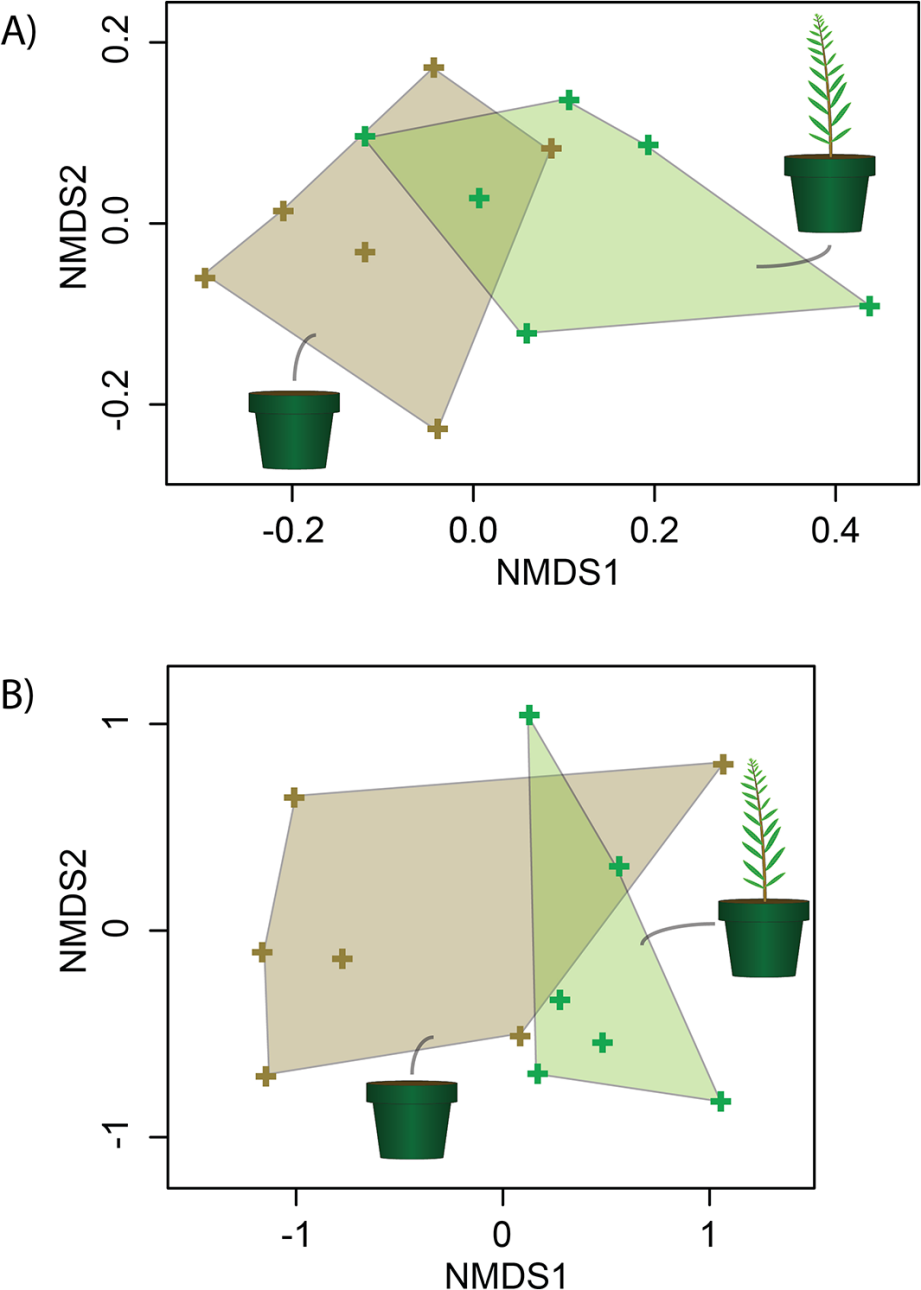
Global influence of *S*. *purpurea* on marker microbial gene expression. Two-dimensional solutions to ordinations, conducted using the non-metric multidimensional scaling (NMDS) method, of global microbial oxygenase transcript abundances measured in bulk contaminated soil samples (brown crosses and shaded areas) and rhizosphere soil samples (green crosses and shaded areas). A) Microbial community-wide expression of the 10 selected genes. B) Taxonomically resolved expression of the 10 selected genes (70 gene-taxon combinations).

**Fig 2:**
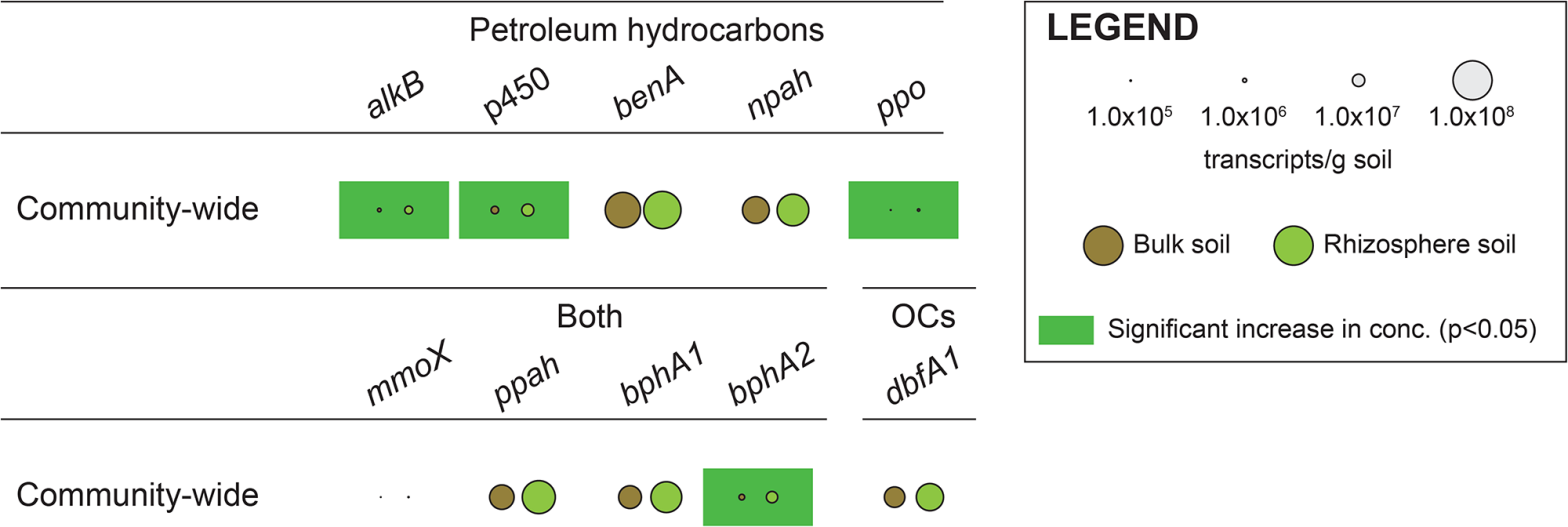
Gene-specific assessment of *S*. *purpurea*’s influence. Community-wide transcript abundance of selected microbial oxygenase genes in bulk and rhizosphere soil samples.

The areas delimited by bulk and rhizosphere soil samples overlapped in the 2-dimensional solution to the NMDS ordination conducted with microbial community-wide oxygenase transcript abundances (stress = 0.07, Fig 1). Nevertheless, their distinct positions clearly demonstrate that *S*. *purpurea* had an effect on the global expression of the associated genes. These results were explained by the fact that all 10 transcript categories were detected in both soil types (i.e. bulk and rhizosphere), but that the abundances per g of soil of those encoding for alkane 1-monooxygenase (5.77×105 ± 3.07×105 vs. 2.83×106 ± 1.55×106, P < 0.01), biphenyl 2,3-dioxygenase small subunit (1.46×106 ± 9.84×105 vs. 5.92×106 ± 4.01×106, P < 0.01), cytochrome P450 monooxygenase (2.57×106 ± 5.16×105 vs. 6.67×106 ± 1.54×106, P < 0.01), and laccase/polyphenol oxidase (2.05×104 ± 2.31×104 vs. 2.45×105 ± 1.92×105, P = 0.03) genes were all more abundant in rhizosphere soil (Fig 2).

Transcript abundances also varied between genes independently of soil types. Two distinct groups were observed: a high expression group that included genes encoding for benzoate 1,2-dioxygenases, naphthalene dioxygenases, aromatic ring hydroxylating dioxygenases, biphenyl 2,3-dioxygenase large subunits, and dibenzofuran 4,4a-dioxygenase large subunits (all in the 1.92–6.36×107 transcripts per g of soil range), and a low expression group that included genes encoding for alkane 1-monooxygenases, cytochrome P450 monooxygenases, laccase/polyphenol oxidases, methane monooxygenase component A alpha chains, and biphenyl 2,3-dioxygenase large subunits (all in the 103–106 transcripts per g of soil range).

### Taxonomic identity of xenobiotic-degrading microorganisms stimulated by *S*. *purpurea*

**Fig 3:**
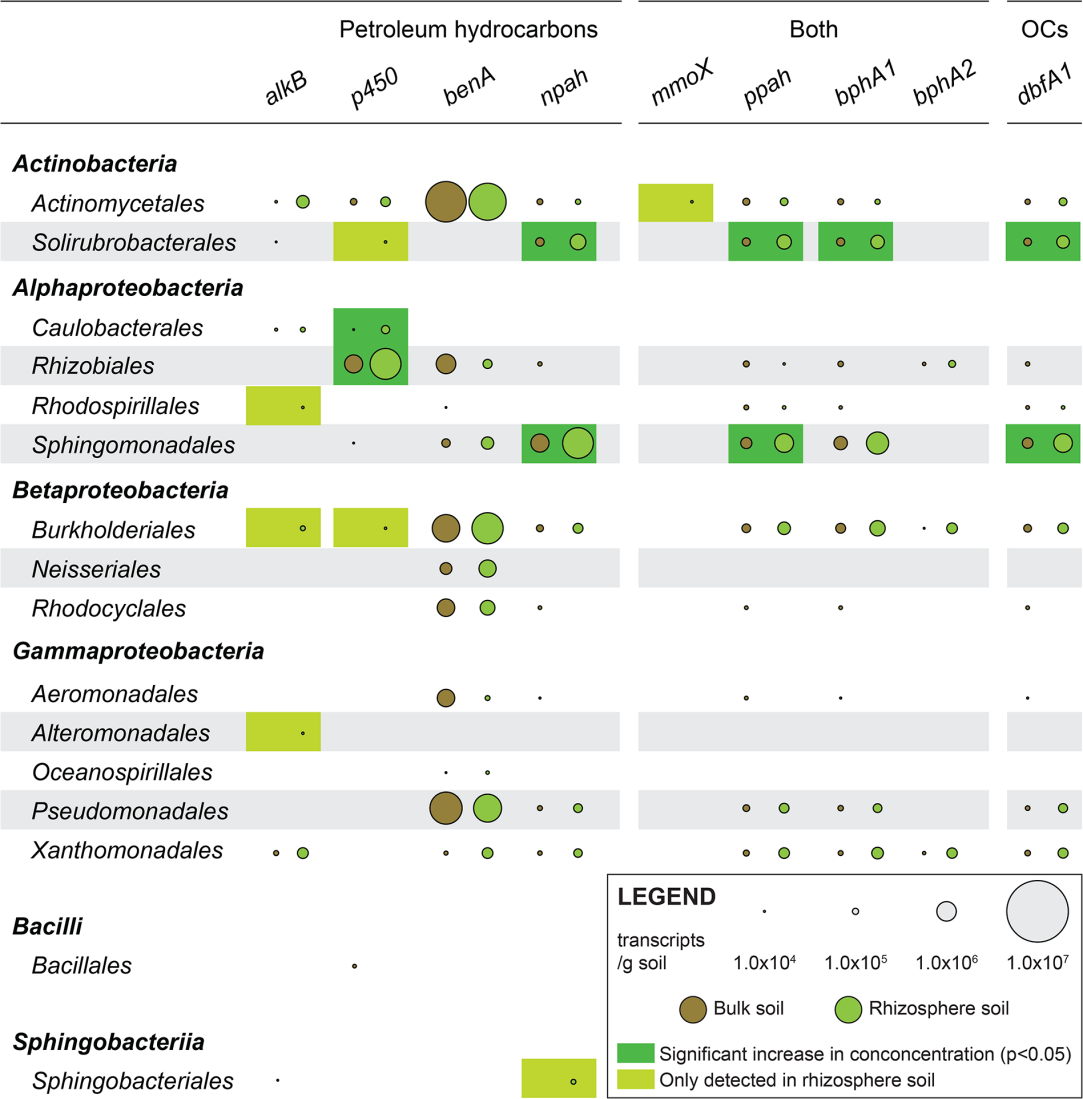
Taxonomically resolved assessment of *S*. *purpurea*’s influence. Transcript abundance of identified gene-taxon combinations in bulk and rhizosphere soil samples.

Approximately 50% of the transcripts that carried oxygenase genes could be assigned a taxonomic qualifier at the level of order or lower (i.e. from order- to strain-level). All gene searches contributed to this pool, with the exception of those that targeted laccases/polyphenol oxidases, whose associated transcripts remain unidentified. Collectively, the taxonomically classified transcripts belonged to 16 different orders, all of which were bacterial (Fig 3). Overall, members of the orders *Actinomycetales* (8/10 protein families), *Burkholderiales* (8/10 protein families), *Rhizobiales* (7/10 protein families), and *Xanthomonadales* (7/10 protein families) were detected with the highest number of gene searches. In opposition, members of the orders *Alteromonadales*, *Neisseriales*, *Oceanospirillales*, *Bacillales*, and *Sphingobacteriales* were infrequently observed (1/10 protein family each). Similarly, the selected oxygenase genes ranged in the diversity with which they were distributed among detected orders. Benzoate 1,2-dioxygenase alpha subunits (11/16 groups), aromatic ring hydroxylating dioxygenases (10/16 groups), biphenyl 2,3-dioxygenase large subunits (10/16 groups), and dibenzofuran 4,4a-dioxygenase large units (10/16 groups) were the most widely spread, and methane monooxygenase component A, alpha chain (1/16 groups) at the most narrowly distributed.

Areas delimited by bulk and rhizosphere soil samples also partly overlapped in the 2-dimensional solution to an NMDS ordination conducted with taxonomically resolved oxygenase transcript abundances (stress = 0.12, Fig 1). Yet, *S*. *purpurea*’s global effect on the combined expression of the 70 detected gene-taxon combinations was once more clearly demonstrated. As with community-wide data, these results were explained by the fact that 36 transcript categories were detected in similar abundances in both soil types, and 34 were significantly more abundant in one or the other (Fig 3). The categories that were more abundant in rhizosphere soil could be classified in two groups: those present in small abundances in rhizosphere soil and completely absent from bulk soil, and those significantly more abundant in rhizosphere soil. The first group included the methane monooxygenase component A alpha chains of *Actinomycetales*, the cytochrome P450 monooxygenases of *Solirubrobacterales*, the alkane 1-monooxygenases of *Rhodospirillales*, the alkane 1-monooxygenases and cytochrome P450 monooxygenases of *Burkholderiales*, the alkane 1-monooxygenases of *Alteromonadales*, and the naphthalene dioxygenases of *Sphingobacteriales*. The second category included the alkane 1-monooxygenases of *Actinomycetales* (1.66×104 ± 2.57×104 vs. 4.15×105 ± 1.80×105, P < 0.01), the naphthalene dioxygenase (1.89×105 ± 1.72×105 vs. 6.46×105 ± 2.75×105, P < 0.01), aromatic ring hydroxylating dioxygenases (1.72×105 ± 1.66×105 vs. 5.44×105 ± 1.56×105, P < 0.01), biphenyl 2,3-dioxygenase large subunits (1.73×105 ± 1.54×105 vs. 4.82×105 ± 1.28×105, P < 0.01), and dibenzofuran 4,4a-dioxygenase large subunits of *Solirubrobacterales* (1.55×105 ± 1.45×105 vs. 4.21×105 ± 7.24×104, P < 0.01), the cytochrome P450 monooxygenases of *Caulobacterales* (8.39×103 ± 2.06×104 vs. 1.76×105 ± 1.19×105, P = 0.02), the cytochrome P450 monooxygenases of *Rhizobiales* (8.60×105 ± 6.34×105 vs. 2.52×106 ± 1.26×106, P = 0.02), and the naphthalene dioxygenases (8.69×105 ± 7.30×105 vs. 2.49×106 ± 1.77×106, P = 0.03), aromatic ring hydroxylating dioxygenases (3.26×105 ± 2.21×105 vs. 9.26×105 ± 6.73×105, P = 0.03), and dibenzofuran 4,4a-dioxygenase large subunits of *Sphingomonadales* (2.97×105 ± 2.11×105 vs. 9.18×105 ± 6.21×105, P = 0.02). Several of the transcripts that belonged to these gene-taxon combinations (i.e. that carried a gene whose expression was significantly more abundant in the rhizosphere for the associated order) were assigned a taxonomic qualifier at the species and/or strain level (Table 2). Gene-taxon combinations whose transcripts were only detected in bulk soil included the alkane 1-monooxygenases of *Solirubrobacterales*, the naphthalene dioxygenases and dibenzofuran 4,4a-dioxygenase large subunits of *Rhizobiales*, the benzoate 1,2-dioxygenase alpha subunits and biphenyl 2,3-dioxygenase large subunits of *Rhodospirillales*, the cytochrome P450 monooxygenases of *Sphingomonadales*, the naphthalene dioxygenases, aromatic ring hydroxylating dioxygenases, biphenyl 2,3-dioxygenase large subunits, and dibenzofuran 4,4a-dioxygenase large subunits of *Rhodocyclales*, the naphthalene dioxygenases, aromatic ring hydroxylating dioxygenases, biphenyl 2,3-dioxygenase large subunits, and dibenzofuran 4,4a-dioxygenase large subunits of *Aeromonadales*, the cytochrome P450 monooxygenases of Bacillales, and the alkane 1-monooxygenases of *Sphingobacteriales*.

**Table 2:** Species- and strain-level taxonomic assignments. Species- and strain-level taxonomic assignments obtained for transcripts associated with gene-taxon combinations that were significantly more abundant in rhizosphere soil than in bulk soil.

Taxa						
	alkb	p450	npah	ppah	bphA1	dbfA1
*Actinobacteria*						
*Solirubrobacterales*						
*Conexibacter woesei* DSM 14684			X	X	X	X
*Actinomycetales*						
*Aeromicrobium marinum* DSM 15272	X					
*Frankia* sp. EuI1c	X					
*Micromonospora lupini* str. Lupac 08	X					
*Nocardioides* sp. JS614	X					
*Alphaproteobacteria*						
*Caulobacterales*						
*Caulobacter* sp. K31		X				
*Phenylobacterium zucineum* HLK1		X				
*Rhizobiales*						
*Bradyrhizobiaceae* bacterium SG-6C		X				
*Bradyrhizobium diazoefficiens* USDA 110		X				
*Bradyrhizobium* sp. ORS 375		X				
*Bradyrhizobium* sp. S23321		X				
*Bradyrhizobium* sp. STM 3809		X				
*Bradyrhizobium* sp. STM 3843		X				
*Parvibaculum lavamentivorans* DS-1		X				
*Sphingomonadales*						
*Sphingobium japonicum* UT26S				X		X
*Sphingobium yanoikuyae*				X		X
*Novosphingobium* sp. PP1Y			X	X		X
*Sphingomonas paucimobilis*				X		X
*Sphingomonas polyaromaticivorans*			X	X		X
*Sphingomonas* sp. Ibu-2				X		X
*Sphingomonas* sp. LH128			X			

The orders *Solirubrobacterales* and *Sphingomonadales* topped the list of taxa that showed the highest numbers of induced rhizosphere gene expression. *Actinomycetales*, *Burkholderiales*, *Caulobacterales*, *Rhizobiales*, *Rhodospirillales*, *Alteromonadales*, and *Sphingobacteriales* were also in this category. On the other hand, the gene expression attributed to *Neisseriales*, *Rhodocyclales*, *Aeromonadales*, *Oceanospirillales*, *Pseudomonadales*, *Xanthomonadales*, and *Bacillales* were either only detected in bulk soil or not significantly different between soil types.

## Discussion

As an early step towards the improvement of willow-based soil cleanup applications, we aimed to explore *S*. *purpurea*‘s microbially assisted rhizoremediation capabilities from a functional metatranscriptomic perspective. To determine if the plant influences the expression of 10 microbial oxygenase genes that are markers of coveted xenobiotic degradation, transcripts were sorted from sequence datasets previously generated with bulk and rhizosphere contaminated soil [14], and an NMDS ordination and gene-specific statistical comparisons were performed. An apparent rhizosphere effect characterized by the enhanced expression of genes encoding for alkane 1-monooxygenases, cytochrome P450 monooxygenases, laccase/polyphenol oxidases, and biphenyl 2,3-dioxygenase small subunits was detected. Based on the known substrates of the selected enzymes, and on a characterization of the studied contamination [14], these observations could be manifestations of *S*. *purpurea*-microbe xenobiotic degradation systems that target C4-C16 alkanes, fluoranthene, anthracene, benzo(a)pyrene, biphenyl, and/or PCBs. This interpretation is consistent with the demonstrated rhizoremediation capabilities of willows [9, 33, 35], but it also expands the list of common soil contaminants that may be cleaned up with these trees.

To identify microorganisms that are likely involved in targeted *S*. *purpurea*-microbe xenobiotic degradation systems, each of the singled out transcript was then assigned a parent taxon. These analyses highlighted members of 16 bacterial orders, all of which are known to comprise species that possess authentic copies, or putative homologs, of the searched genes. Indeed, the Fungene databases that were used to assign taxonomic qualifiers to transcripts are composed of sequences gathered through searches for gene sequence homology [41]. Many of the identified gene-taxon combinations are also supported by functional characterizations. For example, several members of the order *Actinomycetales* make use of the genes assigned herein to the group to degrade alkanes (e.g. *Rhodococcus erythropolis*, *Rhodococcus rhodochrous*, *Mycobacterium* sp.HXN-1500) [44, 47, 59], aromatic hydrocarbons (e.g. *Rhodococcus* strain NCIMB12038, *Rhodococcus* sp. I24) [48, 52], and PCBs (e.g. *Rhodococcus erythropolis*) [56]. The same goes for members of the orders *Burkholderiales* (e.g. *Burkholderia cepacia*, *Burkholderia* sp. DBTI, *Ralstonia eutrophus*, *Ralstonia* sp. U2) [44, 48, 59], *Sphingomonadales* (e.g. *Novosphingobium aromaticivorans*, *Sphingobium yanoikuyae*, *Sphingomonas* sp. strain RW1) [47, 56, 60], *Bacillales* (e.g. *Bacillus megaterium*) [47], and *Pseudomonadales* (e.g. *Pseudomonas putida*, *Pseudomonas* sp. JS42, *Pseudomonas pseudoalcaligenes*) [48, 56, 60]. These observations suggest that the conducted taxonomic assignments were accurate. Yet, gene-taxon combinations that haven’t been formally demonstrated were also identified. Some of these claims are supported by phylogenetic analyses (e.g. presence of alkane 1-monooxygenase genes in the genome of species belonging to the orders *Solirubrobacterales* and *Rhodospirillales*) [61], or circumstantial evidence (e.g. presence of proteins belonging to members of the orders *Rhizobiales* and *Rhodocyclales* in naphthalene- and fluorine-degrading mixed cultures) [62], and may therefore be genuine. But some could nevertheless be the result of incorrect gene and/or taxonomic assignments. For example, members of the order *Actinomycetales* do not, to our knowledge, possess authentic methane monooxygenases. These are rather features of aerobic methylotrophs, a group that includes members of the orders *Methylococcales*, *Rhizobiales*, and *Methylacidiphilales* [63]. Additional analyses will have to be conducted to determine the significance of the latter results in the context of our study.

A second NMDS ordination, this time conducted with the transcript abundances of all identified gene-taxon combinations, confirmed that some of the plant’s influence was captured in our taxonomically-resolved data. Subsequent statistical comparisons between soil types demonstrated that the expression of alkane 1-monooxygenase and cytochrome P450 monooxygenase genes by members of the orders *Actinomycetales*, *Rhodospirillales*, *Burkholderiales*, *Alteromonadales*, *Solirubrobacterales*, *Caulobacterales*, and *Rhizobiales* was higher in rhizosphere soil than in bulk soil, and thus that they may be involved in the associated C4-C16 alkane- and fluoranthene-degrading systems. These observations strengthen and clarify the previously hypothesized rhizoremediation roles of members of the orders *Rhodospirillales*, *Burkholderiales*, and *Rhizobiales* [14].

However, the microorganisms whose expression of laccase/polyphenol oxidases or biphenyl 2,3-dioxygenase small subunits was stimulated could not be identified. Although laccases are widely distributed in nature [64], their aromatic degradation capabilities are best demonstrated in the fungal homologs [53]. Yet, no taxonomic qualifier could be assigned to the transcripts that carried these genes, and that despite the presence of fungal sequences is the database used for their identification. The specificity of willow-fungi detoxification systems that exploit these enzymes may partly explain *Salix* spp.’s selection of *Pezizomycetes* or *Dothideomycetes* representatives in the highly contaminated soil of our study’s associated field site [65]. Thus, the identity of these transcripts' parent organisms remains an important open question.

Taxa-specific gene expression enhancements that were not visible at the community level were also detected. They could be signs of *S*. *purpurea*-microbe xenobiotic degradation systems that harness the naphthalene/aromatic ring hydroxylating dioxygenases of *Solirubrobacterales*, *Sphingomonadales*, and *Sphingobacteriales* representatives to degrade various aromatic hydrocarbons (e.g. BTEX, acenaphthylene, anthracene, fluorene, methylnaphthalene, naphthalene, pyrene), the biphenyl 2,3-dioxygenases of *Solirubrobacterales* representatives to degrade biphenyl/PCBs, and the dibenzofuran 4,4a-dioxygenases of *Solirubrobacterales* and *Sphingomonadales* representatives to degrade chlorinated dibenzofuran. Although factors that explain these constrained stimulations could be multiple (e.g. specialized stimulation mechanisms that only affect some taxa, competition for process-limiting nutrients between xenobiotic degraders, variable enzymatic affinity for substrate, temperature/pH affecting the activity of various taxa), it should be noted that all the affected genes were highly expressed in both soil types at the community-level. We hypothesize that this situation may have therefore been caused by rhizosphere concentrations of stimulating exudate components that were proportionally low compared to those of the potentially impacted contaminants. These *S*. *purpurea*-microbe xenobiotic degradation systems would have therefore been active, but their global signature would have been overwhelmed by that of plant-independent microbial xenobiotic degradation activities.

Many of the taxa identified herein as probable *S*. *purpurea* rhizoremediation partners have also been implicated in other such associations. For example, members of the orders *Actinomycetales*, *Rhizobiales*, and *Burkholderiales* appear to participate in the alkane 1-monooxygenase- and cytochrome P450 monooxygenase-driven rhizoremediation of alkanes by ryegrass (*Lolium multiflorum*) and birdsfoot trefoil (*Lotus corniculatus*) [27]. Similarly, members of the order *Actinomycetales* have been associated with petroleum hydrocarbon rhizoremediation by various grasses and legumes [29, 30]. The ability to stimulate these microorganism' xenobiotic degradation gene expression can therefore be seen as a sign of a plant's rhizoremediation potential, and thus be used to screen other species that are candidates for the development of new soil cleanup applications.

However, the involvement of *Solirubrobacterales* and *Sphingomonadales* members in hydrocarbon rhizoremediation has never been reported, even if representatives of the former taxon have been detected in soil contaminated with petroleum products [66], and if the latter contains many well known hydrocarbon degraders. Our results also contrast with those presented by studies that aimed to identify plant PCB rhizoremediation partners, as *Solirubrobacterales* representatives are not known to play such roles. Studies that focused on *Salix* spp. rather point towards *Rhodococcus*, *Pseudomonas*, *Sphingobacterium*, and *Burkholderia* species [12, 28, 31]. Work that has assessed other plants’ capabilities points to members of the orders *Rhizobiales*, *Pseudomonadales*, and *Actinomycetales* [28, 31, 32]. If real, this plant-specific variability is likely linked in part to that of the underlying root exudates [67, 68], which reinforces the idea suggesting that rhizoremediation-driven exudate optimization needs to be precisely tailored for the plant-microbe systems that perform the coveted xenobiotic degradation.

Our study provides preliminary information in an area that is crucial for the bioengineering of willow-based hydrocarbon rhizoremediation. It opens the door for the development of models that reproduce the underlying detoxification systems, and thus for the identification of process-limiting exudate characteristics. As they potentially target a broad range of common contaminants, the *S*. *purpurea*-microbe systems that putatively involve members of the orders *Solirubrobacterales* and *Sphingomanadales* should be first in line for laboratory testing. The stimulation of *Sphingomonadales'* aromatic ring hydroxylating dioxygenase (*ppah*) gene expression is, for example, consistent with a salicylic acid-triggered benzo(a)pyrene degradation. Indeed, the phenolic acid is a well-known component of willow exudates; it enhances *Sphingomonas yanoikuyae*'s degradation of the aromatic hydrocarbon in the laboratory [69]; benzo(a)pyrene degradation is one of the known substrates of aromatic ring hydroxylating dioxygenases; and *S*. *yanoikuyae* is one of the close relatives of microorganisms that were identified by the taxonomic assignment of *ppah* transcripts. Albeit indirect, similar evidence unites salicylic acid exudation and a potential *Actinomycetales* methane monooxygenase-stimulated phenanthrene degradation [70, 71]. Salicylate has also been shown to be an inducer of biphenyl 2,3-dioxygenase-driven PCB degradation by members of the order *Actinomycetales* in contexts that are similar to that of our study [72–74]. Its potential implication in the stimulation of *Solirubrobacterales* members' biphenyl 2,3-dioxygenase gene expression should therefore be tested. The role of flavonoid, terpenoid, and other phenolic acid emissions in *S*. *purpurea*-microbe xenobiotic degradation systems should also be evaluated, as some of these compounds can stimulate microbial hydrocarbon and polychlorinated biphenyl degradation under laboratory conditions [69, 70, 75–79]. The plant’s release of carbohydrates, amino acids, and other low molecular weight organic acids are also of interest, as these compounds are broadly used microbial nutrient and energy sources and notorious stimulants of global soil microbial activity [80, 81].

The release of root exudates is one of the factors that contribute to the enhanced microbial xenobiotic degradation that characterizes many rhizoremediation applications. Indeed, several studies have demonstrated that other parameters, such as soil oxygenation by plant roots, also contribute significantly to the process [72–74]. Nevertheless, the laboratory optimization of rhizoremediation-proficient exudates has the potential to be a game changer for phytoremediation. Many important innovations have stemmed from our ability to harness and improve plant-microbe interactions that are analogous to those that are triggered by these chemical mixtures [82–84]. We propose that such optimization would probably be a solution to mitigate the competitive woes of rhizoremediation, which could open a market that likely exceeds 30 billion US dollars [2].
